# Genetic and Metabolic Diversity of Cyanobacteria on the Rock–Water Interface in Mountainous Ecosystems

**DOI:** 10.1021/acs.est.5c05763

**Published:** 2025-09-16

**Authors:** Juliana Oliveira, Francesca Pittino, Christoph Scheidegger, Sabine Fink, Elisabeth M.-L. Janssen

**Affiliations:** † Biodiversity and Conservation Biology, 28500 Swiss Federal Research Institute for Forest Snow and Landscape Research (WSL), Birmensdorf 8903, Switzerland; ‡ Department of Environmental Chemistry, 28499 Swiss Federal Institute of Aquatic Science and Technology (EAWAG), Dübendorf 8600, Switzerland; § Department of Earth and Environmental Sciences, 9305 University of Milano-Bicocca, Milan 20126, Italy

**Keywords:** metabarcoding, toxin, anabaenopeptin, microcystin, cyanopeptolin, *Tintenstrich*, lichen

## Abstract

*Tintenstrich* communities are specialized lithic biofilms dominated by free-living cyanobacteria, also occurring in lichen associations, forming a unique ecological interface between rock environments and aquatic habitats in mountainous areas. To better understand their composition and genetic and metabolic potential, we analyzed 207 samples from the Swiss Alps and Jura Mountains. We determined how key environmental factors shaped cyanobacterial abundance, assessed whether these communities harbor genes for toxin biosynthesis, characterized their taxonomic composition at the family and genus level, and evaluated the actual occurrence of cyanotoxins and other bioactive metabolites. Cyanobacterial abundance proved to be influenced by factors such as elevation, exposure, and their interaction with siliceous rock substrata. Targeted PCR and Sanger sequencing revealed the presence of toxin-encoding genes, particularly for *ndaF*/*mcyE* fragments, which may encode microcystin and/or nodularin biosynthesis, while specific genes for microcystins, anatoxins, and cylindrospermopsins were rather rare. Metabarcoding analysis identified 11 cyanobacterial families, with Chroococcaceae, Nostocaceae, and Leptolyngbyaceae being the most abundant. Complementary high-resolution mass spectrometry confirmed the occasional presence of nodularins and microcystins, alongside more frequent detection of other bioactive peptides such as anabaenopeptins and cyanopeptolins. Overall, these findings provide the most comprehensive insight to date into *Tintenstrich*-associated cyanobacteria, underscoring their environmental significance given their genetic and metabolic potential.

## Graphical abstract

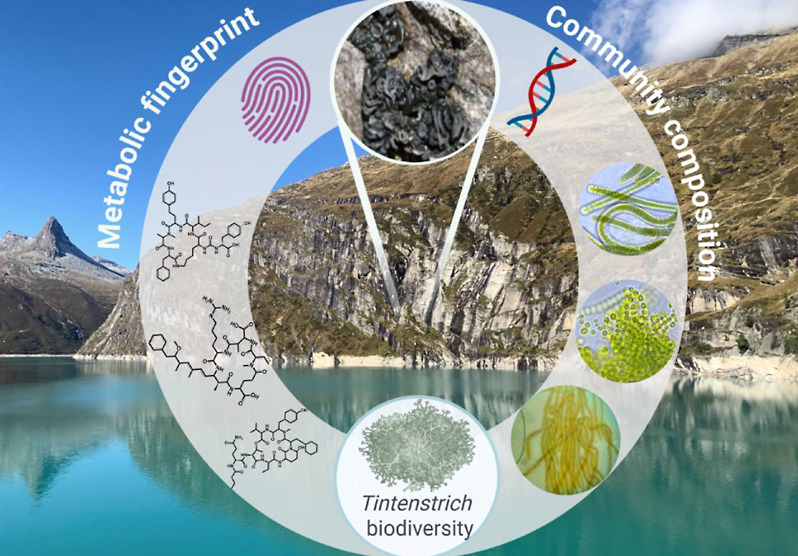

## Introduction

Mountain ecosystems hold highly specialized communities adapted to often hostile environmental conditions,^1,2^ and many aspects regarding their biodiversity remain to be explored. One kind of these specialized communities are subaerial biofilms, which spread over large areas of rock surfaces in semiaquatic regions, forming dark morphologies called *Tintenstrich*, the German term for ink-stripe.^3^ These communities inhabit a wide range of altitudes, spanning from lowland to nival areas,^4–7^ and are well-known for their ability to thrive under harsh environmental conditions.^8–10^ *Tintenstrich* are widely distributed in mountain systems^11^ and are primarily composed of free-living cyanobacteria. These can also occur in symbiotic associations with fungi (e.g., cyanolichens), forming part of lithic ecosystems and creating different microhabitats. *Tintenstrich* are found on various rock substrata (e.g., calcareous and siliceous substrate) and mainly in three ecological niches: epilithic (on the rock surface), hypolithic (on the underside of rocks), and endolithic (a few millimeters beneath the surface where light can still penetrate),^12,13^ making them well-suited to survive the challenging conditions found in mountainous environments.

Mountainous areas endure intense stressors including strong UV radiation, temperature fluctuations, limited nutrient resources, and restricted water availability on exposed substratum reported from temperate, arid, and tropical climate zones worldwide.^14^ In *Tintenstrich* environments, for instance, the inclination and orientation of rock surfaces, i.e., exposure, may promote stress conditions due to high radiation. In fact, exposure significantly affects rock wall temperatures, ranging from exceptionally high in daytime during summer and extremely low at night and during winter, which can impact cyanobacterial growth and pigment composition.^15^ In addition, Swiss mountain regions and alpine lakes are predominantly oligotrophic environments,^16^ characterized by limited nutrient availability that impacts cyanobacterial growth and development. Consequently, nutrient limitations are expected to influence the presence and abundance of *Tintenstrich*.

Water availability constitutes one of the most critical stressors in this context. As *Tintenstrich* are part of semiaquatic ecosystems, they promote a crucial connection between terrestrial and aquatic habitats in which their formation is linked to seasonal water run-offs on steep rocks.^3^ During dry seasons, *Tintenstrich* are often found as desiccated biofilms on the surfaces of rock walls;^17^ however, they can swiftly recover upon hydration events, such as rainfall and snowmelts,^18^ a trait not only attributed to free-living cyanobacteria present in the *Tintenstrich* but also to terrestrial cyanolichens.^19^ While a comprehensive taxonomic analysis of mountainous *Tintenstrich* communities is not available to date, cyanolichens in urban settings on building walls can resemble *Tintenstrich*-like communities, with *Chroococcidiopsis*, *Gloeocapsopsis*, *Nostoc*, *Brasilonema*, *Scytonema,* and *Hapalosiphon* reported among the genera present.^20^

The ability of cyanobacteria to regulate and adapt in dynamic and selective environments is also linked to the production of secondary metabolites with diverse ecological functions.^22^ These metabolites not only contribute to stress tolerance and competition but also play a crucial role in shaping the microbial community structure and ecosystem dynamics. Cyanobacteria are often known for the production of toxic secondary metabolites, and in aquatic systems, these toxins can pose a significant concern for both environmental and public health.^23^ Therefore, the World Health Organization (WHO) established threshold values for four cyanobacterial toxins in drinking water and recreational water quality guidelines, namely, for microcystin-LR, anatoxin-a, cylindrospermopsin, and saxitoxin.^24^ The presence of the WHO-recognized toxins has also been reported in cyanolichens: for instance, the biosynthetic gene involved in microcystin production, *mycE*, was identified in 12% of 803 samples from terrestrial cyanolichen, having microcystins directly detectable in 5% of all samples.^25^ Therein, significant concentrations of nodularins, structurally and toxicologically related to microcystins, have been reported in lichen symbiosis with *Nostoc*.^25^ On the other hand, mass spectrometry analyses on cyanobacteria-rich loess crusts, targeting microcystins, cylindrospermopsin, saxitoxins, and other bioactive metabolites, did not detect any of the mentioned toxins.^26^ Free-living and symbiotic cyanobacteria are also known to coproduce a multitude of other bioactive metabolites along with those recognized as toxins by the WHO.^27,28^ Until the end of 2024, a total of 3085 secondary metabolites from cyanobacteria were identified (CyanoMetDB, Version03, 2024).^29,30^ The co-occurrence of other bioactive metabolites together with the WHO-recognized toxins in aquatic environments has long been, and continues to be, a subject of investigation.^31–35^ However, no comprehensive assessment of toxin-encoding genes, toxins, or other bioactive metabolites from cyanobacteria in *Tintenstrich* lithic habitats is currently available.

The present study focuses on assessing the taxonomic diversity and potential for bioactive metabolites production in cyanobacteria from *Tintenstrich* communities inhabiting mountainous environments from the Swiss Alps and Jura Mountains. We aimed at (1) identifying cyanobacterial abundance patterns in relation to key environmental gradients, including rock substrate, elevation, exposure, and microhabitat; (2) determining whether these communities harbor genetic potential for toxin biosynthesis, given the wide distribution and, therefore, the ecological relevance of *Tintentrich;* and (3) characterizing their taxonomic composition at the family and genus level, both to address the lack of comprehensive assessments for these communities and to identify likely contributors previously described as toxin producers or known to harbor toxin biosynthesis genes. In addition, considering the well-established capacity of cyanobacteria to produce bioactive and toxic compounds, we further (4) determined the occurrence of cyanotoxins and related metabolites within these communities. This final objective allowed us to bridge the gap between the genetic potential and actual toxin production in our samples. By integration of ecological and functional approaches, this research enhances our understanding of the environmental drivers influencing cyanobacterial communities in lithic habitats and establishes a foundation for future studies to investigate the broader ecological significance and environmental implications of cyanobacteria in these ecosystems.

## Methods

### Sampling

The sampling campaign took place in spring 2021 and included 19 regions in the Swiss Alps and Jura Mountains, from which 207 rock samples were obtained with sterilized hammer and chisel, as previously described in Pittino et al. 2024 ^13^ (Figure 1). The sampling design aimed at providing a comprehensive overview of *Tintenstrich*, including both free-living cyanobacteria and those in association with lichens (cyanolichens). To achieve this, multiple samples were collected from different sections of the same rock wall as well as from distinct rock walls across all studied regions (details of the number of samples obtained per region are given in Table S1). Samples from each site were analyzed separately to investigate the natural variability of *Tintenstrich* systems and to assess how different environmental gradients influence genetic and metabolic end points. For all samples, we documented four specific environmental factors and their gradients at the collection sites including elevation, ranging from 370 to 3500 m above sea level (m.a.s.l.), and classified as colline (370–900 m.a.s.l.), montane (1000–2150 m.a.s.l.), subalpine (1670–2450 m.a.s.l.), and alpine (2050–3500 m.a.s.l.); rock substrate as carbonate (e.g., limestone) and siliceous (e.g., mica schist); three categories of microhabitat types based on the morphology of the rock surfaces (contiguous = same 3D morphology, Figure S1a; mixed = condition in between, Figure S1b; highly fragmented = heterogeneous surface scattered among green-algal lichen and bryophytes, Figure S1c); and exposure. Table S3 lists the number of samples obtained for each environmental condition. All rock samples were stored at −20 °C until processing.

**Figure 1:**
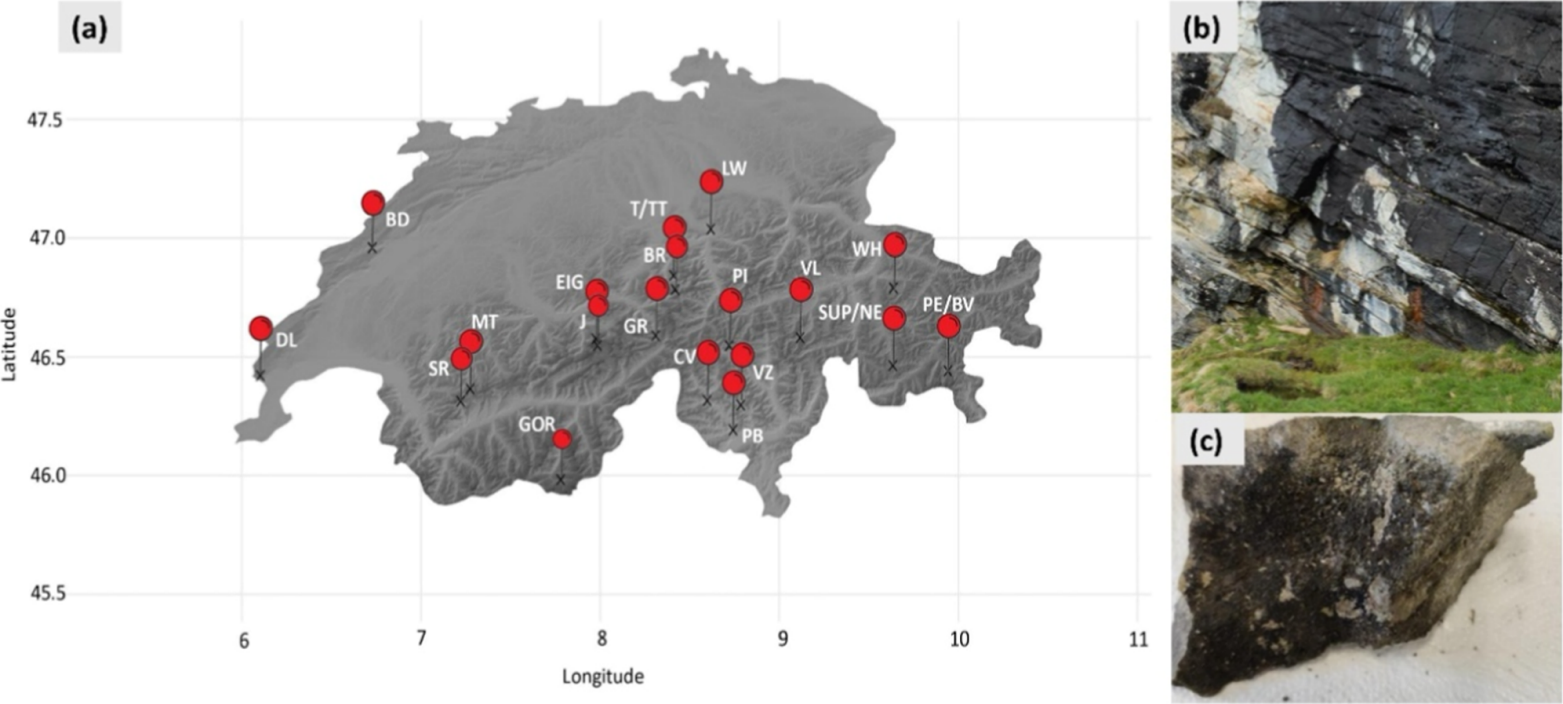
(a) Map of sampling sites in the mountainous areas of Switzerland showing the 19 sampling regions where a total of 207 rock samples with *Tintenstrich* biomass were collected (BD, Brot Dessous; DL, La Dôle; SR, Scex Rouge; MT, Mittaghore; EIG, Eiger; GR, Grimsel Pass; J, Jungfrau; T/TT, Engelberg; BR, Bärschwendi; LW, Lauerz; PI, Piora Valley; GOR, Gornergrat; CV, Cevio; PB, Ponte Brolla; VZ, Verzasca Valley; VL, Vals; SUP/NE, Bivio; WH, Weisshorn; PE/BV, Morteratsch); and exemplary images (b) of a rock wall with dark *Tintenstrich* features and (c) of a recovered rock sample for genetic and metabolic analysis.

### DNA Extraction and Metabarcoding

Biomass was scraped off from rock samples from the top 3–4 mm with the help of a sterile chisel and a hand-held rotary power tool (Dremel 8220-1, Bosch NL) and lyophilized. DNA extraction was performed from 0.25 g of lyophilized biomass following the manufacturer’s instruction of the DNeasy PowerSoil Pro Kit (QIAGEN, protocol dated May 2019), with the deviation of using two metal beads in a sterile 2 mL Eppendorf instead of the PowerBead Pro Tube and adding a 2 min vortexing in dry conditions as a first step. A first polymerase chain region (PCR) was performed on the V4–V5 hypervariable region of the 16S rRNA to check for DNA quality and inhibition using the original DNA extract (undiluted) and both 1:10 and 1:100 dilutions. The PCR mixture was composed of 7.5 μL of JumpStart REDTaq ReadyMix (Sigma-Aldrich), 0.75 μL of each primer 515F and R926 ^13^ (primer concentration of 10 μM; for primer information, see Table S2), 4.5 μL of nanopure water, and 1.5 μL of DNA. The PCR program was as follows: 3 min of initial denaturation at 95 °C, 28 cycles of 45 s at 95 °C, 45 s at 50 °C, 90 s at 72 °C, and a final extension of 5 min at 68 °C. PCR amplification was checked on an agarose gel (2%) for the presence of bands. The dilutions of samples showing a clear band after the first PCR were selected to proceed with sequencing. At this point, a second PCR was performed with KAPA HiFi Hotstart ReadyMix (Roche) and 10 μM of the two primers 515F and 806R to amplify the V4 hypervariable region of the 16S rRNA according to Pittino et al. 2024 and a final volume of 2 × 20 μL per sample.^13^ Primers were modified with Illumina adapters, a shift and a linker according to Kozich et al. 2013 ^36^ for the 515F and according to Caporaso et al. 2012 ^37^ for the 806R. The PCR program was as follows: 3 min of initial denaturation at 95 °C, 28 cycles of 45 s at 95 °C, 45 s at 58 °C, 45 s at 72 °C, and a final extension of 5 min at 72 °C. PCR products were sent to the NGS Platform of the Institute of Genetics at Bern University (Bern, Switzerland) for sequencing with the MiSeq Illumina platform (Illumina, Inc., San Diego, CA) using a 2 × 250 bp paired-end protocol. The amplified product length was 290 bp. For all 207 samples, demultiplexed reads were clustered in Amplicon Sequences Variants (ASVs) with DADA2 ^38^ and taxonomically classified with the SILVA database (v138.1).^39^ Results of the same samples regarding order level classification were previously reported.^13^

### Statistical Analyses for Genetic Data

Before any processing, chloroplast sequences were excluded. Further analyses were conducted using only ASVs classified as cyanobacteria at the phylum level. The data set was normalized prior to the removal of noncyanobacterial sequences, by converting absolute counts into relative abundances (%) across all detected taxa, while retaining the full resolution of ASVs and samples without averaging. To analyze the genetic amplicon sequence data at the Cyanobacteria phylum level, generalized linear models (GLMs) were applied testing various distributions (binominal, Poisson, and quasi-Poisson). However, due to poor model fit, indicated by high deviations of residuals, GLMs were deemed unsuitable, and for this reason, their results are not reported. Instead, linear mixed-effects models were implemented using maximum likelihood estimation, incorporating environmental variables (elevation, rock type, microhabitat, and exposure; Table S3) as predictors and having a random intercept variance of 0.05 to account for variation among sampling areas, which proved to be an adequate fit (Figure S2). We analyzed both the individual effects of each variable and their combined effects in all possible pairwise, three-way, and four-way combinations (Table S4), thereby accounting for the full range of interactions among the variables, for which subsequent pairwise comparisons were conducted using *t* tests with Satterthwaite’s approximation, as implemented in the *lmerTest* package.^40^ Mapping plots were generated to visualize abundance responses incorporating interactions between one or more statistically significant environmental factors. Specifically for the interaction between elevation and exposure, mapping was performed using only the extreme exposure values (lowest and highest percentiles). Two curves were plotted to summarize the data and better explain its relationship (Figure S3a). Analyses were conducted in *Rstudio* (version 4.2.2) using the packages *lme4*, *lmerTest*, and *MASS*, after having used the packages *tidyverse* and *dplyr* to transform the data.^40–43^ For analysis at the family and genus level, the same modeling approach was applied; however, none of the models yielded an adequate fit, making reliable interpretation at the family or genus level unfeasible. To enable further exploration at the family level, despite this limitation, abundance data of each detected amplicon sequence per sample was renormalized to sum 100%, allowing family level abundances to be expressed relative (%) to the cyanobacterial phylum. Sequences that did not match any entry in the SILVA database with a score of 0.8 or higher were classified as “not matched”, and sequences matching strains without genus-level classification are termed “not assigned”. To facilitate interpretation, abundance graphs were organized by rock type, and pairwise comparisons of the relative abundance of each cyanobacterial family were performed using pairwise Tukey’s test. Principal component analysis (PCA) was conducted using the *prcomp* function from the base R *stats* package.^44^ The variables used for the loadings included the relative abundance of all different families per sample, excluding the family category “not matched”. The influence of elevation, rock type, microhabitat type, and exposure on both principal components (PC1 and PC2) was assessed through a permutational multivariate analysis of variance (PERMANOVA). Data were first checked for variance inflation factor using the *car* package^45^ to detect potential overfitting, and PERMANOVA was performed using the *adonis* function under the *vegan* package.^46^ Visualizations of the PCA results were generated using the *ggplot2*.^47^ Both bar and violin plots were also generated with *ggplot2* but considering average abundance of ASVs and displayed at family level data based on rock type (carbonate and siliceous) to simplify the presentation of results. Posthoc tests using Tukey’s method^48^ were conducted to assess pairwise differences in the structure of cyanobacterial families. For the most abundant families, samples were further analyzed for differences at the genus level, and plots were generated likewise.

### Sanger Sequencing for Toxin-Encoding Genes

The presence of cyanotoxin-specific genes and 16S rRNA specific fragments of cyanobacteria was further investigated through Sanger sequencing. We targeted synthetase gene fragments indicative of microcystins and/or nodularins (*ndaF*/*mcyE*, HEPF), also specific genes for microcystins (*mcyE*), anatoxins (*anaF*), and cylindrospermopsin (*cyrJ*), and checked for the presence and classification of cyanobacteria using targeted 16S rRNA gene regions amplified with specific primer pairs. The respective PCR primers and conditions are specified in Table S2. Classification is shown only for samples in which both forward and reverse sequences could be successfully aligned (alignment details are given in Text S1; classification results are given in Supporting Information). All PCR products were checked for the presence of bands on 2% agarose gel, purified, and subsequently sent to Microsynth (Balgach, Switzerland) for Sanger sequencing of both forward and reverse sequences. The raw data was checked and aligned using the software MEGA11 ^49^ (https://www.megasoftware.net/). However, for five specific samples, it was not possible to obtain sufficiently long and reliable sequences for alignment. As a result, only partial sequence fragments were analyzed. This limitation was exclusively observed in samples containing *cyrJ* genes and in a single sample containing *mcyE* genes, all of which were ultimately excluded from reporting. In contrast, for most samples, the sequences were successfully aligned. The results were checked with BLAST on the NCBI database (https://blast.ncbi.nlm.nih.gov/Blast.cgi) to determine best matches for identification of synthetase genes and cyanobacteria gene fragments.^50^

### Chemical Analysis of Toxins and Metabolites

#### Sample Extraction and Analysis

A subset of 68 samples that tested positive for *mcyE* and *mcyE*/*ndaF* genes in a preliminary PCR analysis was selected for the metabolite screening. Biomass was scraped off from rock surfaces, yielding 0.25–5 g of raw material. Harvested biomass values were highly unbalanced due to variability in the biofilm density of each sample, rock type, and surface morphology. Furthermore, inconsistencies in rock size, influenced by field conditions, posed additional challenges to standardization. For the extraction, 1 mL of methanol was added to the material recovered from the rock samples (20–200 mg dry weight, depending on the biomass available) in FastPrep tubes (reaction tube, 2 mL, PP, 10/45 MM) with glass beads (approximately 343 mg, 150–212 μm, Sigma). All samples were passed through a cell disruptor (2 × 20 s at 4 m/s; FastPrep, MP Biomedicals), followed by 15 min of sonication (VWR, Ultrasonic cleaner USC-THD, level 9, 10 °C), and pellets were separated from the supernatant by centrifugation (10,000*g*, 4 °C, 10 min, Megafuge 1.0 R). The supernatant was transferred to a glass vial, the extraction was repeated three times, and supernatants were combined. The extracts were stored at −20 °C until analysis. Extraction efficiency and recovery tests were not performed for *Tintenstrich* biomass as this would require accounting for matrix effects in each of the 209 individual samples. Instead, we focused on a qualitative evaluation, reporting the presence or absence of compounds without pursuing further quantitative analyses. The analysis was performed by high-pressure liquid chromatography (HPLC, Dionex UltiMate3000 RS pump, Thermo Fisher Scientific) coupled to a high-resolution tandem mass spectrometer (HRMS/MS, Exploris 240, Thermo Fisher Scientific). A previously validated method using an automated enrichment and cleanup by online solid phase extraction was used, online SPE (20 mg of Oasis HLB sorbent, 15 μm). Briefly, 600 μL of extract was diluted in 11.4 mL of nanopure water and enriched onto the online SPE system, washed with nanopure water and 20% methanol, eluted with methanol, and automatically diluted with water to refocus on the analytical HPLC column (Kinetex C18, 2.1 × 100 mm, 2.6 μm, Phenomenex, precolumn VanGuard Cartridge, Waters). The mobile phases of the HPLC consisted of (A) nanopure water and (B) methanol, both acidified with formic acid (0.1%), and operated at a flow rate of 255 mL/min, increasing eluent B from 0% to 100% over 25 min. HRMS/MS used electrospray ionization (ESI) with 320 °C capillary temperature, 3.5 kV electrospray voltage, positive ionization mode, full scan from 450 to 1350 *m*/*z* with a nominal resolving power of 120,000 at *m*/*z* 250, 1 × 10^6^ automated gain control (AGC), 100 ms maximal injection time, and 1 ppm mass accuracy. Data-dependent high-resolution product ion spectra were obtained by normalized collision energies for HCD of 15%, 30%, and 45% at a resolving power of 17,500 at 400 *m*/*z*, 5 × 10^4^ AGC, 70 ms maximal injection time, and a 1 *m*/*z* isolation window, triggering data-dependent MS^2^ acquisition using CyanoMetDB (version 02, 2023),^52^ the database of secondary metabolites from cyanobacteria.^29,30,52^ For the analysis of polar metabolites, including anatoxins and cylindrospermopsins, 200 μL of the sample extract was evaporated and resuspended in 150 μL of 5% methanol. From this solution, 100 μL was directly injected into the HPLC system (Waters Atlantis T3, 150 × 3 mm, 3 μm, precolumn VanGuard Cartridge, Waters) at a flow rate of 300 μL/min with eluent B being methanol with 0.1% formic acid and eluent A being nanopure water with 0.1% formic acid and increasing eluent B from 5% to 95% over 25 min. HRMS/MS settings were the same as used for the cyanopeptide analysis with the following differences: the full scan ranged from 100 to 1200 *m*/*z* with a nominal resolving power of 140,000 at *m*/*z* 250. The instrument limit of detection (iLOD, ng/L) and method limit of detection (mLOD, pg/mg) are provided in Table S5 in the Supporting Information.

#### Metabolite Analysis

The subselected 68 samples were screened for metabolites based on the WHO-recognized cyanotoxins and expanded to its derivatives within the same class of metabolites including 330 microcystins, 12 anatoxins, 5 cylindrospermopsins, and 39 saxitoxins. We further included 16 nodularins because they are of comparable toxicological concern as microcystins^53^ and 125 anabaenopeptins as we observed higher frequency of this class of metabolites in our samples by preliminary MS^1^ screening. Nineteen samples that presented a confirmed variant of these classes were further screened for 197 cyanopeptolins, 77 aeruginosins, and 87 microginins if the peak areas were at 10^5^ or higher. The described suspect screening was performed with Skyline 20.1 (MacCoss Lab Software), and in silico fragmentation predictions were used to facilitate compound identification by MS^2^ spectra annotation (MetFrag Web with CyanoMetDB, Version02 2022 database). Predicted and measured spectra were manually evaluated, and the level of confidence was assessed.^54^ Only those cyanopeptides are reported herein that could be identified as one of the following criteria: as *tentative candidate* Level 3 based on exact mass (<4 ppm mass error), accurate isotopic pattern (Skyline idotp value >0.9), and evidence from fragmentation data; as *probable structure*, Level 2C, based on complete fragmentation information confirming building block connectivity but without differentiation between isomers; *probable structure*, Level 2B, based on complete fragmentation and identification of a single compound; *probable structure*, Level 2A, based on complete fragmentation and identification of a single compound matching an available reference spectrum (MassBank Europe or primary literature as cited in CyanoMetDB); and as *confirmed structure*, Level 1, when these parameters were in agreement with available reference standards or bioreagents, whose detection limits were in the low ng/L range (Text S2 and Table S5). To reduce the risk of false positive results, a rigorous verification process was implemented to ensure that all instances of positive identification for a given compound exhibited consistent fragmentation profiles and retention times across the samples. When available, fragmentation was cross-referenced with confirmed structures from cyanobacterial extracts of other species available in the laboratory. The annotation of all reported compounds can be found in the Supporting Information.

## Results and Discussion

### Cyanobacterial Abundance and Environmental Variables

The Cyanobacteria phylum comprises between 17% and 42% of the classified phyla across all 19 sampling regions in the Swiss Alps and Jura Mountains.^13^ Building on that, we first investigated whether patterns in cyanobacterial abundance emerged in association with each of four individual environmental factors and their gradients: rock type, elevation, exposure (compass direction), and microhabitat type (see Statistical Analyses for Genetic Data in the Methods section and Table S3). To assess these relationships, a multilevel correlation model was employed, incorporating the sampling region as a random effect, which significantly improved model fit and suggested that additional region-specific environmental factors, beyond those explicitly tested, may influence cyanobacterial abundance.

Among the five environmental variables examined, elevation, exposure, and rock substrate exerted significant effects on cyanobacterial abundance, both independently and through interactions (Table S4). When considered individually, abundance declined with increasing elevation and with greater exposure toward the west and south (0°, 90°, 180°, and 270°). In contrast, interactive effects showed increased abundance when higher elevations coincided with westward or southward exposures, and when exposure was combined with siliceous rock substrata (for *p*-values and additional statistics, see Table S4 in the Supporting Information).

These patterns are consistent with the well-known sensitivity of cyanobacteria to light and moisture availability, in which both insufficient and excessive light can constrain its development.^56^ Greater exposure, especially on south-facing slopes, often correlates with reduced water availability, a critical factor limiting colonization.^57,58^ Also, microhabitats with shading and rough surfaces can enhance water retention, supporting higher growth and abundance. On the other hand, although elevation has been associated with higher cyanobacterial abundance in some aquatic systems,^55^ the harsher conditions of high mountain environments likely impose stronger selective pressures on lithic communities, leading to the observed overall abundance decline. Previous Antarctic studies also report enriched cyanobacterial communities on siliceous rocks, particularly at lower elevations, underscoring the interactive influence of elevation and substrate type.^59^ In the present study, carbonate rocks may have facilitated subsurface water flow,^60^ whereas the rough textures and microcrevices of siliceous rocks likely enhanced surface water retention, sustaining microbial communities within thin moisture films.

Given that these environmental factors shape the cyanobacterial distribution, it is pertinent to ask whether these patterns are reflected in their functional potential. Cyanobacteria can harbor genes associated with toxin production, linking their observed ecological prevalence to potential environmental impacts. In mountainous ecosystems such as *Tintenstrich*, where cyanobacteria are both abundant and widely distributed, assessing the presence of toxin-related genes provides a direct connection between observed environmental patterns and underlying functional genetic traits.

### Presence of Toxin-Encoding Genes in *Tintenstrich* Cyanobacteria

To assess the genetic basis of the functional role of cyanobacteria in *Tintenstrich*, we employed end-point PCR followed by Sanger sequencing to identify toxin-producing genes in all 207 *Tintenstrich* samples, as each exhibited cyanobacteria-specific fragments. First, PCR analysis revealed the presence of toxin-related biosynthesis genes in the *Tintenstrich* samples, with varying detection rates confirmed by Sanger sequencing (Figure S5).

The *mcyE* gene, associated with microcystin production, was amplified in 17% of the samples by PCR, although only two of them (1%) were validated through sequencing. The *mcyE*/*ndaF* genes, involved in microcystin/nodularin biosynthesis, were detected in 25% of samples by PCR and 20% by Sanger sequencing, totaling 41 confirmed samples. Similarly, the *anaF* gene, involved in anatoxin synthesis, was identified in 34% of samples by PCR but confirmed in only 5% through sequencing, resulting in 10 validated samples. The *anaF* primers used herein may also amplify nonspecific bands, yet other available primers were reported to be at times too specific and therefore risking false negative results especially in complex environmental matrices and samples with low numbers of the target genes.^97^ Due to a high sequence variability of anatoxin biosynthesis genes, nested PCR approaches may prove more effective in future analyses.^97^ Lastly, even though 62 samples (30%) revealed PCR products for the *cyrJ* gene involved in the biosynthesis of cylindrospermopsins, Sanger sequencing confirmed its presence only in four samples (2%). Unlikely the majority of samples reported to contain toxin-encoding genes, these four specific samples and one of those that tested positive for the *mcyE* gene could not be aligned due to their short sequence length and were therefore not reported. Overall, discrepancy between analysis methods of end-point PCR and Sanger sequencing (Figure S5), especially related to *anaF*, *cyrJ*, and *mcyE* genes, could relate to low copy numbers of the environmental samples insufficient for Sanger sequencing confirmation, degraded DNA or mixed PCR products resulting in false-positive amplification identification (bands on the agarose gel referring to other sequences). To evaluate false positive and false negative results, future analysis would need to focus on cloning and sequencing PCR products, which is outside the scope of the presented work.

Overall, the analysis demonstrates the presence of toxin biosynthetic gene sequences in *Tintenstrich* samples from lithic habitats, with a higher prevalence of genes associated with the *mcyE*/*ndaF* biosynthetic pathway, indicating the potential for toxin production in mountain environments. Although BLAST searches provided strain-level matches, the toxin-producing genes could not be confidently linked to any specific cyanobacterial genera or species identified. This limitation arises from the heterogeneous nature of the *Tintenstrich* material, which consists of a complex mixture of cyanobacteria and other microorganisms. Consequently, matches obtained from BLAST may reflect similarities to multiple taxa, and in samples where toxin genes were detected, any direct attribution to particular families or genera could be misleading. For this reason, strain-level BLAST results are presented in the Supporting Information but are not further discussed herein.

### Cyanobacterial Composition in Lithic *Tintenstrich* Communities

Although toxin-producing genes detected in *Tintenstrich* could not be confidently assigned to specific genera, analyzing its community composition provides insight into which taxa may harbor genetic potential for toxin production. With this objective, data in Figure 2 illustrate the abundance of cyanobacteria and of selected cyanobacterial families according to the rock type. Overall, 21.6% to 53.3% of amplicon sequences could not be classified at the family level; nevertheless, 11 cyanobacterial families were identified in *Tintenstrich* samples, with our discussion focusing on the three most abundant ones.

**Figure 2:**
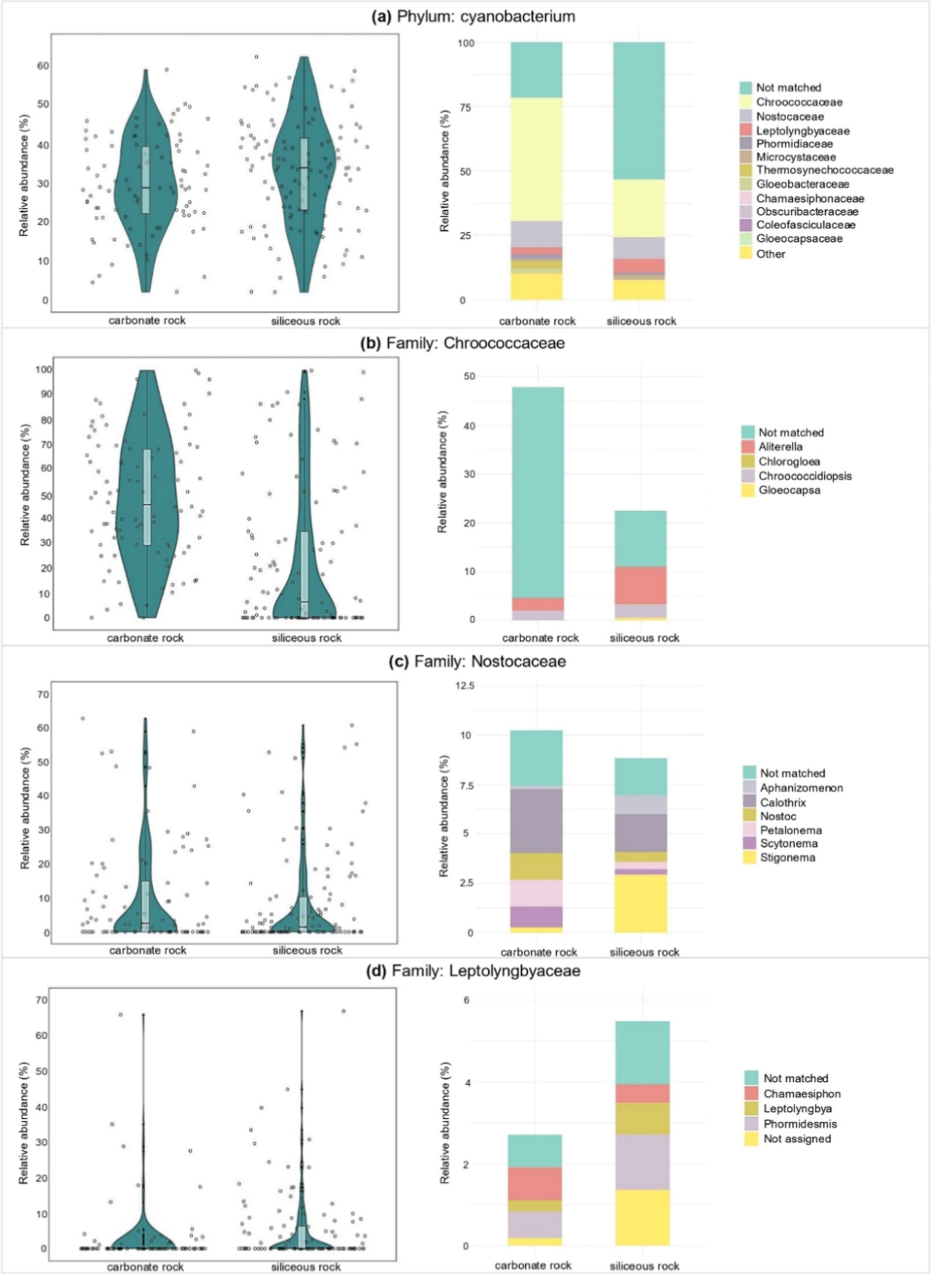
(a) Violin plots show the sum of relative amplicon sequences variants (ASVs) per sample considering the whole cyanobacterial phylum, grouped by carbonate and siliceous rock; bar plots indicate the relative ASV abundance per family identified, averaged, and grouped by rock type. (b–d) Violin plots show the sum of relative ASV abundance per family considering samples within carbonate and siliceous rock; bar plots indicate the relative ASV abundance per genera identified, averaged, and grouped by rock type (carbonate: 92 samples; siliceous: 115 samples). The three most abundant families specified are (b) Chroococcaceae (higher in carbonate rocks, *p* = 3 × 10^–11^), (c) Nostocaceae (higher in carbonate rocks, *p* = 0.015), and (d) Leptolyngbyaceae.

Chroococcaceae represented the most abundant family, with average abundances of 47.9% on carbonate rock and 22.3% on siliceous rock, followed by Nostocaceae with 10.2% and 8.5% and Leptolyngbyaceae with 2.7% and 5.3%, respectively (Figure 2a). Pairwise comparisons showed significantly greater abundances on carbonate rock for Chroococcaceae (*p* = 3 × 10^–11^) and Nostocaceae (*p* = 0.015). Inspecting the abundance of cyanobacterial families, PCA and PERMANOVA indicated that principal component scores were not significantly influenced by the studied environmental variables (Figure S4, Pr­(>*F*) = 0.565).

A high percentage of sequences could not be assigned to a genus for the Chroococcaceae family (11.5% on carbonate and 43.3% on siliceous rock; Figure 2b). Among the resolved taxa, *Aliterella* and *Chlorogloea* remain rarely studied, while *Chroococcidiopsis* and *Gloeocapsa* are well-known lithic colonizers, consistent with our results.^61^ Species traditionally assigned to these genera have been regarded as extremophilic cyanobacteria,^62–72^ although recent phylogenetic work has demonstrated that several strains represent distinct genera,^73^ such as the lineage formally known as “Cold Desert *Chroococcidiopsis*” that has been reclassified as *Aliterella*,^74^ the most abundant classified genus in our data set. However, the production of toxic metabolites by these organisms remains uncharacterized.

Although less dominant, Nostocaceae exhibited a well-resolved taxonomic composition, with only 1.9–2.8% of sequences unclassified at the genus level (Figure 2c). Six genera were identified, including the well-known *Calothrix* *,* commonly reported in freshwater and epilithic habitats,^39,75–77^ as well as *Nostoc* and *Scytonema* *,* known to occur in both aquatic^78,79^ and terrestrial environments,^25,80,81^ highlighting the ecological versatility of rock–water interface ecosystems. Members of this family are known to tolerate harsh environmental conditions,^18,82,83^ and such resilience may be supported by the production of secondary metabolites with adaptive functions.^22,84^ *Nostoc* species are known to synthesize the WHO-recognized hepatotoxins, including microcystin-LR variants,^24,85^ and a structurally related hepatotoxin, nodularin-R.^86^ The detection of Nostocaceae in *Tintenstrich* therefore suggests considerable potential for toxin production.

Leptolyngbyaceae is the third most abundant family (8.2% overall abundance, Figure 2d). Therein, all genera identified were previously reported in both aquatic and terrestrial ecosystems.^87–90^ *Leptolyngbya* species have been reported to also synthesize variants of the hepatotoxin microcystin-LR,^91,92^ and although toxin production in *Phormidesmis* is not documented to date, a strain of *Phormidesmis priestleyi* possesses the genetic potential for microcystin biosynthesis.^93^ Even though less prevalent, grouped under the “other” category (Figure 2a), the families Phormidiaceae (1.02–1.82%) and Microcystaceae (0.88–0.92%) both contain genera frequently associated with the production the WHO-recognized toxins from the chemical classes of anatoxins and microcystins, respectively (Figure 2a).^68,94,95^

Overall, the cyanobacterial families and genera identified in our study suggest potential for toxin production. However, since the detected toxin-encoding genes could not be directly assigned to specific taxa, we subsequently focused on metabolite detection to assess the potential expression of these genes.

### Presence of Toxins and Other Bioactive Metabolites in *Tintenstrich* Cyanobacteria

To investigate whether we can identify the presence of actual toxins and other secondary metabolites, we further inspected a subset of 68 samples that tested positive for *mcyE* and *mcyE*/*ndaF* genes by PCR analysis using mass spectrometry. Analogous to the analysis of toxin-encoding genes, we first focused the metabolite analysis on those compounds that the WHO recognized as toxins. Within each compound class, many variants exist, and we screened for 330 microcystins, 16 nodularins, 12 anatoxins, 5 cylindrospermopsins, and 39 saxitoxins. Cylindrospermopsins, anatoxins, and saxitoxins were not identified in any *Tintenstrich* samples; therefore, we will focus our further discussion on microcystins, nodularins, and other bioactive metabolites.

#### Microcystins and Nodularins

Among the 68 samples analyzed, microcystin variants were detected in only two *Tintenstrich* samples. Of those, one corresponded to a sample that tested positive for the *mcyE* gene by PCR but did not show a match by Sanger sequencing, while the other was associated with a sample confirmed to contain *mcyE*/*ndaF* genes, linked to both microcystin/nodularin biosynthesis. The identified microcystins were variants of MC-(4H)­YR and MC-Lhar in a sample from Vals (VL, siliceous rock, nonfragmented microhabitat, alpine habitat at 1957 m.a.s.l., southeast exposure) and a variant of MC-RR in a sample from Bivio (NE, siliceous rock, mixed microhabitat, montane habitat at 1950 m.a.s.l., northwest exposure) (Figure 3; Table S6; Supporting Information). A previous study on cyanobionts of 803 terrestrial lichen from 13 different countries report similar observations, where 12% of all samples presented the biosynthetic gene *mycE* for microcystin production, but microcystins themselves were only detectable in 5% of all samples.^25^ Here, the biomass obtained from the *Tintenstrich* samples was limited, and false negative detections are possible when concentrations fell below the instrument’s limit of detection.

**Figure 3:**
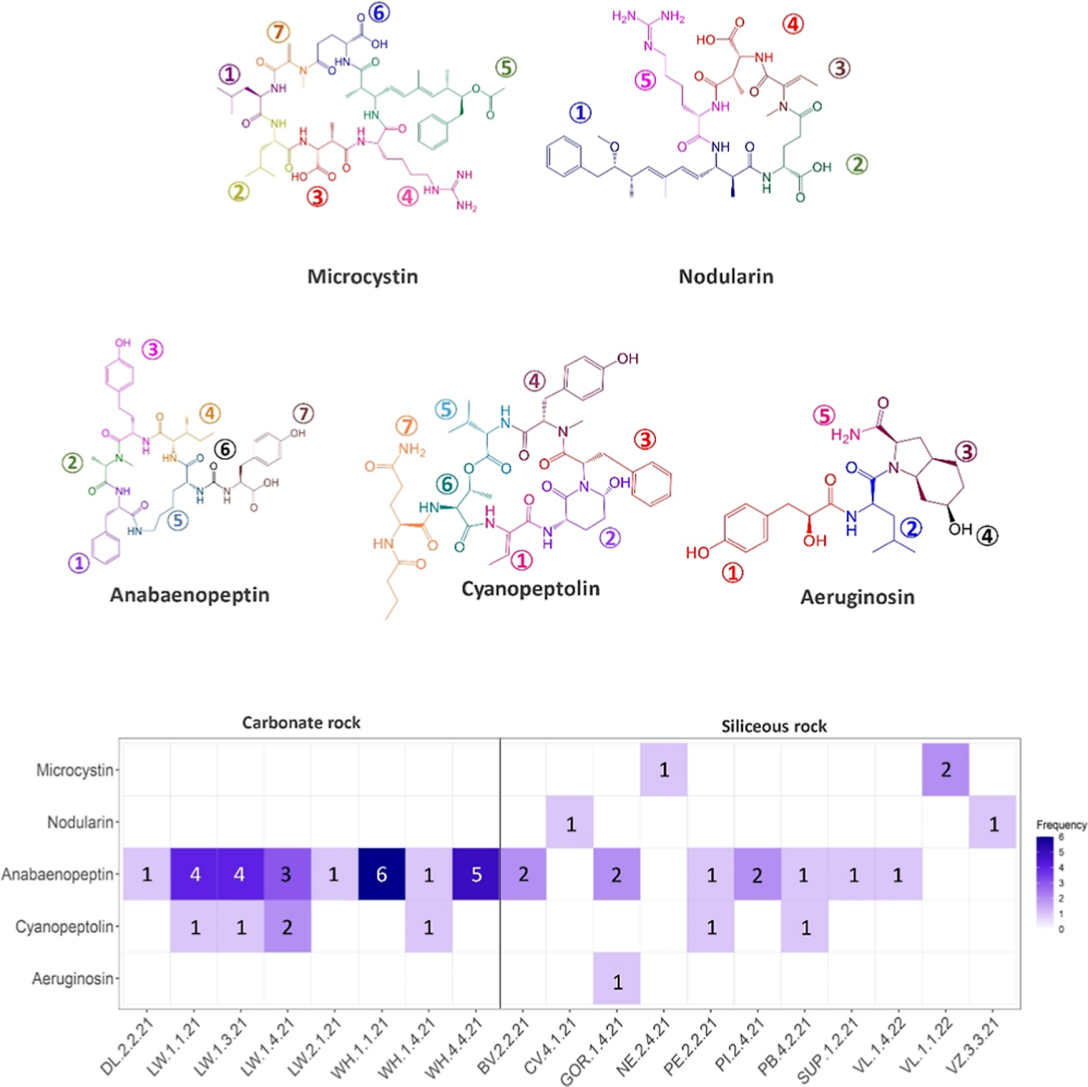
Representative structures of metabolites identified in *Tintenstrich* samples including [d-Leu1,ADMAdda5]­MC-Lhar for microcystins, NOD-Har for nodularins, oscillamide Y for anabaenopeptins, somamide B for cyanopeptolins, and aeruginosin EI461; numbered and colored moieties indicate the different peptide building blocks that can vary across variants within each class (all variants identified are listed in Table S6). The heatmap below illustrates the frequency of different variants of each class, distinguishing carbonate and siliceous rock substrata samples; numbers inside the cell indicate the number of different variants identified (DL, La Dôle; LW, Lauerz; WH, Weisshorn; PE/BV, Morteratsch; CV, Cevio; GOR, Gornergrat; SUP/NE, Bivio; PB, Ponte Brolla, PI, Piora Valley; VL, Vals; VZ, Verzasca Valley). Among the 68 samples analyzed, 19 contained one or more known cyanopeptides, whereas no known metabolites were detected in 44 samples (data not shown).

Among the 40 samples confirmed to contain the *mcyE* **/** *ndaF* genes, only two tested positive for nodularins by mass spectrometry, mirroring the low detection rate observed for microcystins. A NOD-Har variant was identified in a sample from Verzasca Valley (VZ, siliceous rock, mixed microhabitat, colline habitat at 900 m.a.s.l., east exposure) and in a second sample from Cevio (CV, siliceous rock, mixed microhabitat, colline habitat at 620 m.a.s.l., northwest exposure) (Figure 3; Table S6; Supporting Information).

Although microcystins/nodularins were not frequently identified in the *Tintenstrich* samples, the prevalence of toxin-encoding genes suggests that their biosynthesis remains possible and may also reflect concentrations below the analytical detection limits of this study. These observations motivate further investigation on the environmental conditions that trigger nodularin and microcystin production in lithic habitats.

#### Other Bioactive Metabolites

In the initial MS^1^ screening of the selected 68 samples for the respective precursor ions, the class of anabaenopeptins, with 125 different variants known to date, stood out with most tentative hits and was prioritized for verification by MS^2^ annotation. Compared to the WHO-recognized toxins, anabaenopeptins were more frequently identified in 15 out of 68 samples (22%, Figure 3; Table S6; Supporting Information). Their occurrence was evenly distributed between both siliceous and carbonate rock across 9 sampling regions in La Dôle (1 sample), Lauerz (4 samples), Weisshorn (3 samples), Morteratsch (2 samples), and single samples at Bivio, Gornergrat, Ponte Brolla, Piora Valley, and Vals. Carbonate rock samples from Lauerz (LW) and Weisshorn (WH) exhibited the highest frequency of detected metabolites, and up to 6 different anabaenopeptin variants co-occurred. *Nostoc*, *Phormidium*, *Microcystis*, and *Aphanizomenon* are among known genera producing anabaenopeptins.^51,52,103–110^ While these genera were frequently detected across *Tintenstrich* samples, they occurred only in 3 out of 15 samples that tested positive for anabaenopeptins, suggesting that other cyanobacteria may also take part in the production of this class of metabolites. Although anabaenopeptins are not recognized by the WHO as toxins critical for drinking and recreational water quality, these metabolites induce protease inhibition, in which ecological ramifications are attributed to protective effects, for instance, against predation, regulation of cyanobacterial growth, and bioaccumulation within organisms.^113^ The frequent detection of anabaenopeptins in *Tintenstrich* communities may indicate that they also play a major role in these specialized ecosystems.

In a final step, the metabolite screening was further expanded to include 197 cyanopeptolins, 77 aeruginosins, and 87 known microginins for those 19 samples that tested positive for at least one metabolite discussed above. Only one undefined isomer of aeruginosin, aeruginosin EI461-298B, was detected in a sample from the region of Gornergrat (GOR), and no evidence was found for the presence of any microginins. However, cyanopeptolins were identified in 6 samples, including the regions of Lauerz and Weisshorn on carbonate rock, as well as Morteratsch and Ponte Brolla on siliceous rock (Figure 3; Table S6; Supporting Information). The known producers of cyanopeptolins are *Microcystis*, *Nostoc*, *Scytonema*, and *Leptolyngbya*,^114–117^ all of which also occurred in these *Tintenstrich* samples. Like anabaenopeptins, cyanopeptolins are not included in the WHO guidelines but present bioactive properties.^118–124^ From an ecological perspective, three distinct cyanopeptolin variants have shown to cause lethal effects in zebrafish larvae by disrupting nervous system function, and extracts containing only cyanopeptolins exhibited levels of toxicity comparable to extracts containing microcystins.^100^ These observations highlight the ecological significance of cyanopeptolins within the *Tintenstrich* ecosystem.

### Implications

In this study, we demonstrate that abundance patterns in cyanobacterial communities from *Tintenstrich* are influenced by key environmental factors such as elevation, exposure, and their interactions with siliceous rock. While increased levels of elevation and exposure alone tend to reduce cyanobacterial abundance, their combined effects, particularly with siliceous substrate, promoted abundance, suggesting a context-dependent response. In any case, *Tintenstrich* cyanobacteria are widely distributed across the Swiss mountain regions and are now recognized as being highly abundant.

Genes responsible for the biosynthesis of several toxins were detected, with specific genes encoding for anatoxins, cylindrospermopsin, and microcystins rarely occurring, while gene fragments indicative of microcystins and/or nodularins (*ndaF*/*mcyE*) were more frequently observed. Given the potential health risks associated with these toxins, as outlined in the WHO water quality guidelines, the presence of toxin-producing genes in *Tintenstrich* cyanobacteria underscores the need for further investigation into their ecological role and potential risks in these lithic environments, especially at the terrestrial–aquatic interface.

*Tintenstrich* samples from the Swiss mountain regions were found to be highly diverse, yet a substantial proportion of sequences could not be confidently assigned to known families. This limitation likely stems from multiple factors, the foremost being the constraints of reference databases, which may not fully encompass lineages adapted to highly specialized environments such as *Tintenstrich*. The SILVA database used in this study provides extensive coverage of cyanobacterial taxa and is widely employed in cyanobacterial research, representing one of the most comprehensive resources available. Consequently, the presence of a notable fraction of unclassified sequences may reflect the underrepresentation of mountain biofilm-associated cyanobacteria in existing databases.

Five distinct classes of metabolites were identified in our samples, in which anabaenopeptins and cyanopeptolins were frequently detected through chemical analysis of the biomass, while microcystins, nodularins, and aeruginosins were only sporadically observed. These findings present a temporal snapshot of metabolite production in these habitats at the sampling time. Although anabaenopeptins and cyanopeptolins are not currently classified as toxic to humans by the WHO, they are potent inhibitors of enzymes involved in key metabolic processes, which may have ecological relevance in *Tintenstrich* habitats.

As a newly studied ecosystem, numerous gaps remain within the *Tintenstrich* cyanobacterial communities, awaiting further investigation. Particularly, the impact and interactions of these communities with other ecosystem components are not explored, for example, the ecological roles and interactions of *Tintenstrich* with primary consumers at the base of the local food web. Also, potential effects of toxic cyanobacteria on downstream water resources via snowmelt, rainwater, or rockslides remain unknown. This study presents a first comprehensive assessment of the taxonomic diversity of cyanobacteria in *Tintenstrich* communities and their capacity to produce toxins and other bioactive metabolites across a broad geographic range in the Swiss mountain regions, offering foundation for further research.

## Supplementary Material







## Data Availability

The genetic data generated in this study have been deposited in the NCBI SRA repository and are publicly accessible under accession number PRJNA1095436 (https://www.ncbi.nlm.nih.gov/sra/PRJNA1095436).

## Notes

The documented identification of metabolite structures by MS^2^ spectra annotation (XLSX)
Detailed results obtained from Sanger sequencing (XLSX)
Materials; PCR amplification and Sanger sequencing; number of samples collected per sampling region; list of genes sequenced with Sanger sequencing with their primer names and forward and reverse sequences; number of samples categorized based on four environmental parameters: rock type; summary results from linear mixed model applied to cyanobacterial phyla abundance in *Tintenstrich* samples; reference standards and bioreagents used in the metabolite analysis; cyanobacterial metabolites identified in cyanobacterial biomass from *Tintenstrich* samples; examples of *Tintenstrich* microhabitats; diagnostic plots for linear mixed models (LMMs) applied on cyanobacterial phyla abundance in all 207 *Tintenstrich* samples; visualizations depict the effects of environmental variable interactions on cyanobacterial abundance based on linear mixed-effects models; principal component analysis (PCA) performed on family-level data across; all 207 *Tintenstrich* samples; and matrix for toxin-encoding genes detected in *Tintenstrich* samples (PDF)
