# Supplementary material for: Genetic and Metabolic Diversity of Cyanobacteria on the Rock–Water Interface in Mountainous Ecosystems
Source: Environ Sci Technol. 2025 Sep 16;59(38):20595–607. doi: 10.1021/acs.est.5c05763 (PMC12489977; doi:10.1021/acs.est.5c05763)
Supplement: Supplementary file 3 [file es5c05763_si_001.pdf]

Genetic and metabolic diversity of cyanobacteria on the rock-water interface in mountainous ecosystems

Juliana Oliveira,[1,2] Francesca Pittino,[1,3] Christoph Scheidegger[1], Sabine Fink,[1*] Elisabeth M.-L. Janssen[2*]

[1]Biodiversity and Conservation Biology, Swiss Federal Research Institute for Forest Snow and Landscape Research (WSL), Birmensdorf, 8903, Switzerland.

[2]Department of Environmental Chemistry, Swiss Federal Institute of Aquatic Science and Technology (EAWAG), Dübendorf, 8600, Switzerland.

[3]Department of Earth and Environmental Sciences, University of Milano-Bicocca, Milan, 20126, Italy

**co-corresponding authors*
Email: elisabeth.janssen@eawag.ch
Email: Sabine.fink@wsl.ch

Supporting Information

The electronic supporting information contains 12 pages including 2 texts, 6 tables, 5 figures.

Supplementary text information

Text S1. **Materials. PCR amplification and Sanger sequencing.** PCR amplification was performed following the methodology described in the Methods section of the main text. Some additional information is provided below:

Cyanobacteria-specific 16S primers were used (Table S2), with two forward primers pooled in the final dilution to achieve a final concentration of 10 µM, then applied as a single primer. The PCR program consisted of an initial denaturation at 95°C for 3 minutes, followed by 35 cycles of 1 minute at 94°C, 60°C, and 72°C, and a final extension at 68°C for 5 minutes.

For the detection of toxin-encoding genes, the number of PCR cycles was increased (details given below) due to the use of environmental samples. A positive control was included in the gel electrophoresis (3 µL per lane).

To detect microcystins and nodularins, HEPF primers targeting the *mcyE*/*ndaF* genes were used, along with specific primers for *mcyE* (Table S2). PCR conditions included initial denaturation at 95°C for 3 minutes, followed by 40 cycles of 1 minute at 94°C, 61°C, and 72°C, with a final extension at 68°C for 5 minutes. The amplified product length was 470 bp for the *mcyE*/*ndaF*, and 810 bp for *mcyE*.

For *anaF* gene detection, *atxoa* primers were used (Table S2), with initial denaturation followed by 40 cycles of 30 seconds at 94°C, 65°C, and 72°C, and a final extension at 68°C for 5 minutes. The amplified product length was 460 bp; For the *cyrJ* gene, a specific primer was used (Table S2) under the following conditions: initial denaturation, followed by 40 cycles of 30 seconds at 94°C, 63°C, and 72°C, and a final extension at 68°C. The amplified product length was 562 bp.

Text S2. Materials. Microcystin reference standards MC-LR, MC-YR, MC-RR, MC-LF, MC-LA, MC-LW, MC-LY, and nodularin (all >95% purity by HPLC) were obtained from Enzo Life Science (Lausen, Switzerland) and [D-Asp^3, E-Dhb^7]MC-RR (>95% purity by HPLC) from CyanoBiotech GmbH (Berlin, Germany). Bioreagents for Aeruginosin 98B, Cyanopeptolin A, Cyanopeptolin D, Anabaenopeptin A, Anabaenopeptin B, and Oscillamide Y (all >90% purity by HPLC) were obtained from CyanoBiotech. Aerucyclamide A was obtained as purified bioreagent in dimethyl sulfoxide by Prof. Karl Gademann (University Zurich, Switzerland). Reference standards for homoanatoxin-a and dihydrohomoanatoxin-a were obtained from NovaKits and supplied in 0.1% acetic acid solution; anatoxin-a standard was purchased from Enzo Life Sciences. All three standards had a purity greater than 98%.

Supplementary tables

Table S1. Number of samples collected per sampling region.

Sampling region	Number of samples
Bärschwendi, BR	2
Bivio, SUP/NE	19
Brot-Dessous, BD	9
Cevio, CV	8
Eiger, EIG	3
Engelberg, T/TT	22
Gornergrat, GOR	11
Grimsel Pass, GR	6
Jungfrau, J	25
La Dôle, DL	9
Lauerz, LW	8
Mittaghore, MT	12
Morteratsch, PE/BV	13
Piora Valley, PV	10
Ponte Brolla, PB	12
Secx Rouge, SR	9
Vals, VL	4
Verzasca Valley, VZ	10
Weisshorn, WH	15

Table S2. List of genes sequenced with Sanger sequencing with their primer names and forward and reverse sequences.

**Target gene** (target endpoint)	**Sequence length (bp)**	**Forward primer:** name and sequence	**Reverse primer:** name and sequence	**References**
**16S rRNA** (cyanobacteria classification)	**290**	**CYA 781R**— GACTACWGGGGTATCTAATCCCWTT	**CYA 359F**— GGGGAATYTTCCGCAATGGG<br>**CYA106F**— CGGACGGGTGAGTAACGCGTGA	**Allahverdiyeva** (2010); **Nübel** (1997) [1,2]
**mcyE** (microcystins)	**810**	**mcyE F2**— GAAATTTGTGTAGAAGGTGC	**mcyE R4**— AATTCTAAAGCCCAAAGACG	**Maltsev** (2021)[3]
**mcyE/ndaF** (microcystins and nodularins)	**470**	**HEPF** — TTTGGGGTTAACTTTTTTGGGCATAGTC	**HEPR** — AATTCTTGAGGCTGTAAATCGGGTTT	**Casero** (2019)[4]
**anaF** (anatoxins)	**460**	**atxoaf** — TCGGAAGCGCGATCGCAAATCG	**atxar** — GCTTCCTGAGAAGGTCCGCTAG	**Casero** (2019)[4]
**cyrJ** (cylindrospermopsins)	**562**	**cyrJ-F** — TTCTCTCCTTTCCCTATCTCTTTATCT	**cyrJ-F** TTCTCTCCTTTCCCTATCTCTTTATCA	**Ribeiro** (2020)[5]

References

(1) Allahverdiyeva, Y.; Leino, H.; Saari, L.; Fewer, D. P.; Shunmugam, S.; Sivonen, K.; Aro, E.-M. Screening for Biohydrogen Production by Cyanobacteria Isolated from the Baltic Sea and Finnish Lakes. International Journal of Hydrogen Energy 2010, 35 (3), 1117–1127. https://doi.org/10.1016/j.ijhydene.2009.12.030.

(2) Nübel, U.; Garcia-Pichel, F.; Muyzer, G. PCR Primers to Amplify 16S rRNA Genes from Cyanobacteria. Appl Environ Microbiol 1997, 63 (8), 3327–3332. https://doi.org/10.1128/aem.63.8.3327-3332.1997.

(3) Maltsev, Y.; Kezlya, E.; Maltseva, S.; Karthick, B.; Dvořák, P.; Kociolek, J. P.; Kulikovskiy, M. A New Species of the Previously Monotypic Genus Iningainema (Cyanobacteria, Scytonemataceae) from the Western Ghats, India. European Journal of Phycology 2021, 56 (3), 348–358. https://doi.org/10.1080/09670262.2020.1834147.

(4) Casero, M. C.; Velázquez, D.; Medina-Cobo, M.; Quesada, A.; Cirés, S. Unmasking the Identity of Toxigenic Cyanobacteria Driving a Multi-Toxin Bloom by High-Throughput Sequencing of Cyanotoxins Genes and 16S rRNA Metabarcoding. Science of The Total Environment 2019, 665, 367–378. https://doi.org/10.1016/j.scitotenv.2019.02.083.

(5) Ribeiro, M. S. F.; Tucci, A.; Matarazzo, M. P.; Viana-Niero, C.; Nordi, C. S. F. Detection of Cyanotoxin-Producing Genes in a Eutrophic Reservoir (Billings Reservoir, São Paulo, Brazil). Water 2020, 12 (3), 903. https://doi.org/10.3390/w12030903.

Table S3. Number of samples categorized based on four environmental parameters: rock type, elevation, microhabitat type, and exposition.

Environmental factors	Number of samples
**Rock type**	
Siliceous rock	115
Carbonate rock	92
**Environment/elevation** (m.a.s.l.)	
Colline (370-900)	38
Montane (1000-2150)	39
Subalpine (1670-2450)	44
Alpine (2050-3500)	86
**Microhabitat type**	
Contiguous	81
Fragmented	66
Mixed	60
**Exposure**	
North	31
South	45
East	25
West	6
Northeast	26
Northwest	21
Southeast	34
Southwest	19

Table S4. Summary results from linear mixed model applied to cyanobacterial phyla abundance in *Tintenstrich* samples. For model evaluation, the table contains values for Akaike information criterion (AIC), Bayesian Information criterion (BIC), log-likelihood (LogLik), deviance (deviance) and degrees of freedom of residuals (df.resid); The summary statistics of scaled residuals are shown in minimum residual value (Min), first quartile (Q1), median, third quartile (3Q) and maximum residual value (Max); The model output contains the estimate value (presenting the coefficient for the predictor variable in the model, estimate), standard error (std. error), degrees of freedom (Df), t-value, and Pr(>|t|) (p-value).

	**AIC**	**BIC**	**LogLik**	**deviance**	**df.resid**
	1411.7	1498.4	-679.9	1359.7	181
**Scaled residuals**	Min	Q1	Median	3Q	Max
	-3.0541	-0.5054	0.1536	0.6304	1.8086
**Fixed effects**	estimate	std. error	df	t-value	Pr(>|t|)
**(Intercept)**	1.114e+02	8.947e+00	1.584e+02	12.455	< 2e-16
**Siliceous**	-1.807e+01	9.950e+00	1.421e+02	-1.816	0.071
**Elevation**	-1.131e-02	4.555e-03	1.738e+02	-2.483	**0.014**
**Exposition**	-1.415e-01	5.517e-02	2.002e+02	-2.565	**0.011**
**Fragmentationp**	-8.858e+00	1.300e+01	2.065e+02	-0.681	0.496
**Contiguous**	2.077e-02	1.411e+01	2.060e+02	0.001	0.998
**Siliceous:Elevation**	1.057e-02	5.032e-03	1.666e+02	2.100	**0.037**
**Siliceous:Exposition**	1.251e-01	5.879e-02	2.036e+02	2.128	**0.035**
**Elevation:Exposition**	9.297e-05	3.135e-05	2.047e+02	2.965	**0.003**
**Siliceous:Mixed**	1.364e+01	1.465e+01	2.069e+02	0.931	0.352
**Siliceous:Contiguous**	-1.787e+00	1.514e+01	2.049e+02	-0.118	0.906
**Elevation:Mixed**	3.847e-03	6.286e-03	2.070e+02	0.612	0.541
**Elevation:Contiguous**	2.787e-03	6.652e-03	2.056e+02	0.419	0.675
**Exposition:Mixed**	8.024e-02	6.904e-02	2.039e+02	1.162	0.246
**Exposition:Contiguous**	5.055e-02	7.757e-02	1.994e+02	0.652	0.515
**Siliceous:Elevation:Exposition**	-8.291e-05	3.263e-05	2.058e+02	-2.541	**0.011**
**Siliceous:Elevation:Contiguous**	-4.850e-03	7.131e-03	2.063e+02	-0.680	0.497
**Siliceous:Elevation:Contiguous**	-2.223e-03	7.083e-03	2.046e+02	-0.314	0.754
**Siliceous:Exposition:Mixed**	-1.009e-01	7.946e-02	2.070e+02	-1.270	0.205
**Siliceous:Exposition:Contiguous**	-4.614e-02	8.184e-02	2.030e+02	-0.564	0.573
**Elevation:Exposition:Mixed**	-5.326e-05	3.766e-05	2.065e+02	-1.414	0.158
**Elevation:Exposition:Contiguous**	-5.707e-05	3.980e-05	2.034e+02	-1.434	0.153
**Siliceous:Elevation:Exposition:Mixed**	4.246e-05	4.265e-05	2.070e+02	0.996	0.321
**Siliceous:Elevation:Exposition:Contiguous**	5.374e-05	4.127e-05	2.048e+02	1.302	0.194

Chemical analyses

Table S5. Reference standards and bioreagents used in the metabolite analysis with their molecular formula, monoisotopic mass, and dominant precursor ion form with associated mass-to-charge (m/z) ratio, and instrumental limit of detection (iLOD) and method limit of detection (mLOD/dry weight).

Metabolite	Molecular formula	Monoiso-topic mass (Da)	Dominant precursor ion	*m/z* value	iLOD (ng/L)	mLOD (pg/mg DW)
**Cyanopeptides online-SPE LC-HRMS/MS**						
**Aerucyclamide A**	$C_{24}H_{34}N_6O_4S_2$	534.20830	$[M+H]^+$	535.21557	0.5	0.266-2.455
**Anabaenopeptin A**	$C_{44}H_{57}N_7O_{10}$	843.41669	$[M+H]^+$	844.42397	0.1	0.053-0.491
**Anabaenopeptin B**	$C_{41}H_{60}N_{10}O_9$	836.45447	$[M+H]^+$	837.46175	0.2	0.106-0.982
**Oscillamide Y**	$C_{45}H_{59}N_7O_{10}$	857.43234	$[M+H]^+$	858.43962	0.8	0.425-3.928
**Cyanopeptolin A**	$C_{46}H_{72}N_{10}O_{12}$	956.53312	$[M+H]^+$	957.54039	1.2	0.638-5.892
**Cyanopeptolin D**	$C_{48}H_{76}N_8O_{12}$	956.55827	$[M+H]^+$	957.56555	1.1	0.584-5.401
**MC-LR**	$C_{49}H_{74}N_{10}O_{12}$	994.54877	$[M+H]^+$	995.55604	0.1	0.053-0.491
**MC-HilR**	$C_{50}H_{76}N_{10}O_{12}$	1008.56442	$[M+H]^+$	1009.57169	0.2	0.106-0.982
**MC-LA**	$C_{46}H_{67}N_7O_{12}$	909.48477	$[M+H]^+$	910.49205	0.1	0.053-0.491
**MC-LF**	$C_{52}H_{71}N_7O_{12}$	985.51607	$[M+H]^+$	986.52335	0.3	0.159-1.473
**MC-LW**	$C_{54}H_{72}N_8O_{12}$	1024.52697	$[M+H]^+$	1025.53425	4.2	2.231-20.623
**MC-LY**	$C_{52}H_{71}N_7O_{13}$	1001.51099	$[M+H]^+$	1002.51826	0.1	0.053-0.491
**MC-RR**	$C_{49}H_{75}N_{13}O_{12}$	1037.56581	$[M+2H]^{2+}$	519.79018	0.1	0.053-0.491
**MC-YR**	$C_{52}H_{72}N_{10}O_{13}$	1044.52803	$[M+H]^+$	1045.53531	0.2	0.106-0.982
**[D-Asp^3,(*E*)-Dhb^7]MC-RR**	$C_{48}H_{73}N_{13}O_{12}$	1023.55016	$[M+2H]^{2+}$	512.78236	0.1	0.053-0.491
**[D-Asp^3]MC-LR**	$C_{48}H_{72}N_{10}O_{12}$	980.53312	$[M+H]^+$	981.54039	0.2	0.106-0.982
**Nodularin-R**	$C_{41}H_{60}N_8O_{10}$	824.44324	$[M+H]^+$	825.45052	0.1	0.053-0.491
**Polar Metabolites LC-HRMS/MS**						
**Cylindrospermopsin**	$C_{15}H_{21}N_5O_7S$	415.11617	$[M+H]^+$	416.12344	n.d.	n.d.
**Anatoxin-a**	$C_{10}H_{15}NO$	165.11536	$[M+H]^+$	166.12264	n.d.	n.d.
**Homoanatoxin-a**	$C_{11}H_{17}NO$	179.13101	$[M+H]^+$	180.13829	n.d.	n.d.
**Dihydrohomoanatoxin-a**	$C_{11}H_{19}NO$	181.14666	$[M+H]^+$	182.15394	n.d.	n.d.

Table S6. Cyanobacterial metabolites identified in cyanobacterial biomass from *Tintenstrich* samples. The position assigned for each building block can be found in Figure 3 of the main text.

	**Microcystin**	**Molecular formula**	**m/z value**	**1**	**2**	**3**	**4**	**5**	**6**	**7**
**Suspect (level 3)**<br>Although there is evidence for a higher conf. level, samples presented insufficient MS2	[D-Asp3, ADMAdda5]MC-(H4)YR	$C_{52}H_{74}N_{10}O_{14}$	1063.54587	D-Ala	(H4)Tyr	D-Asp	Arg	ADMAdda	D-Glu	Mdha
	D-Leu1,ADMAdda5 MC-Lhar	$C_{54}H_{82}N_{10}O_{13}$	1078.60628	D-Leu	Leu	D-bMe-Asp	Arg	ADMAdda	D-Glu	Mdha
	MC-RR variant<br>(MC-RR)<br>([D-Asp3,D-MeO-Glu6]MC-RR)<br>([(6Z)-Adda5]MC-RR)	$C_{49}H_{75}N_{13}O_{12}$	1038.57309	D-Ala	Arg	D-bMe-Asp<br>D-Asp<br>D-bMe-Asp	Arg	Adda<br>Adda<br>(6Z)-Adda	D-Glu<br>D-MeO-Glu<br>D-Glu	Mdha
	**Anabaenopeptin**	**Molecular formula**	**m/z value**	**1**	**2**	**3**	**4**	**5**	**6**	**7**
**Target (level 1)**	Oscillamide Y	$C_{45}H_{59}N_7O_{10}$	858.43961	Phe	NMeAla	Hty	Ile	D-Lys	CO	Tyr
**Suspect (level 2b)**	Anabaenopeptin 807	$C_{42}H_{61}N_7O_9$	808.46035	Phe	NMeAla	Hty	Ile	Lys	CO	Ile
	Anabaenopeptin SA7	$C_{44}H_{64}N_8O_9$	849.48690	Phe	NMeASn	PNV	Ile	Lys	CO	Ile
	Anabaenopeptin 848	$C_{44}H_{64}N_8O_9$	850.49472	Phe	NMeASn	MeHphe	Ile	Lys	CO	Leu
	Anabaenopeptin 850	$C_{43}H_{63}N_9O_9$	850.49472	Phe	NMeASn	Hphe	Ile	Lys	CO	Lys
	Anabaenopeptin 871	$C_{46}H_{61}N_7O_{10}$	872.45526	Hty	NMeAla	Hty	Ile	Lys	CO	Phe
	Anabaenopeptin 877a	$C_{43}H_{63}N_{11}O_9$	878.48829	Phe	NMeASn	Hphe	Ile	Lys	CO	Arg
	Anabaenopeptin 877b	$C_{45}H_{67}N_9O_9$	878.51345	Phe	NMeASn	EtHphe	Ile	Lys	CO	Lys
	Anabaenopeptin 882	$C_{47}H_{62}N_8O_9$	883.47128	Phe	ASnMe	NMeHphe	Ile	Lys	CO	Phe
	Anabaenopeptin I	$C_{38}H_{61}N_7O_9$	760.46035	Leu	NMeAla	Hyt	Val	D-Lys	CO	Ile
	Nodulapeptin 821	$C_{43}H_{63}N_7O_9$	822.47600	Ser	NMeHphe	Hphe	Ile	Lys	CO	Ile
	Nodulapeptin 823	$C_{42}H_{61}N_7O_{10}$	824.45526	Ser	NMeHty	Hphe	Val	Lys	CO	Ile
	Nodulapeptin 881b	$C_{45}H_{67}N_7O_9S$	882.47937	Met	NMeHty	Hphe	Ile	Lys	CO	Ile
	Nodulopeptin 901	$C_{47}H_{63}N_7O_9S$	902.44807	Met	NMeHty	Hphe	Val	D-Lys	CO	Phe
	Nodulapeptin 807	$C_{42}H_{61}N_7O_9$	808.46035	Ser	NMeHphe	Hphe	Val	Lys	CO	Ile
**Suspect (level 2c)**	Anabaenopeptin 802 or 802a or 802b	$C_{42}H_{58}N_8O_8$	803.44503	Phe	NMeAla	Trp	Ile<br>Leu	Lys	CO	Val
	Anabaenopeptin 863a or SA8	$C_{45}H_{66}N_8O_9$	863.50255	Phe	NMeASn	EtHphe<br>PNL	Ile	Lys	CO	Leu<br>Ile

	Anabaenopeptin 864 or SA4	$C_{44}H_{65}N_9O_9$	864.49780	Phe	NMeASn	MeHphe PNV	Ile	Lys	CO	Lys
	**Cyanopeptolin**	**Molecular formula**	**m/z value**	**1**	**2**	**3**	**4**	**5**	**6**	**7**
**Suspect (level 2b)**	Somamide B	$C_{46}H_{62}N_8O_{12}$	919.45599	Dbh	Ahp	Phe	N-Me-Tyr	Val	O-Thr	Gln-BA
	Cyanopeptolin 983	$C_{48}H_{73}N_9O_{13}$	984.53305	Leu	Ahp	Val	N,O-diMe-Tyr	Val	O-Thr	Gln-Pro-AC
	Micropeptin SF909	$C_{45}H_{63}N_7O_{13}$	910.45566	Gln	Ahp	Leu	N-Me-Tyr	Ile	O-Thr	Hpla
	Cyanopeptolin CP963b or CP963	$C_{49}H_{69}N_7O_{13}$	964.50261	Leu	Ahp	Phe	N,O-Me-Tyr N-Me-Tyr	Val	O-Thr	Glu-BA Asp-HA
	**Aeruginosin**	**Molecular formula**	**m/z value**	**1**	**2**	**3**	**4**	**5**	**6**	**7**
**Suspect (level 2c)**	Aeruginosin EI461 or 298B	$C_{24}H_{35}N_3O_6$	462.25986	L-Hpla	D-Leu	L-diepi-choi L-choi	OH	amide	*	*
	**Nodularin**	**Molecular formula**	**m/z value**	**1**	**2**	**3**	**4**	**5**	**6**	**7**
**Suspect (level 2b)**	NOD-Har	$C_{42}H_{62}N_8O_{10}$	839.46616	Adda	D-Glu	Mdhb	Masp	Har	*	*

4

Supplementary figures

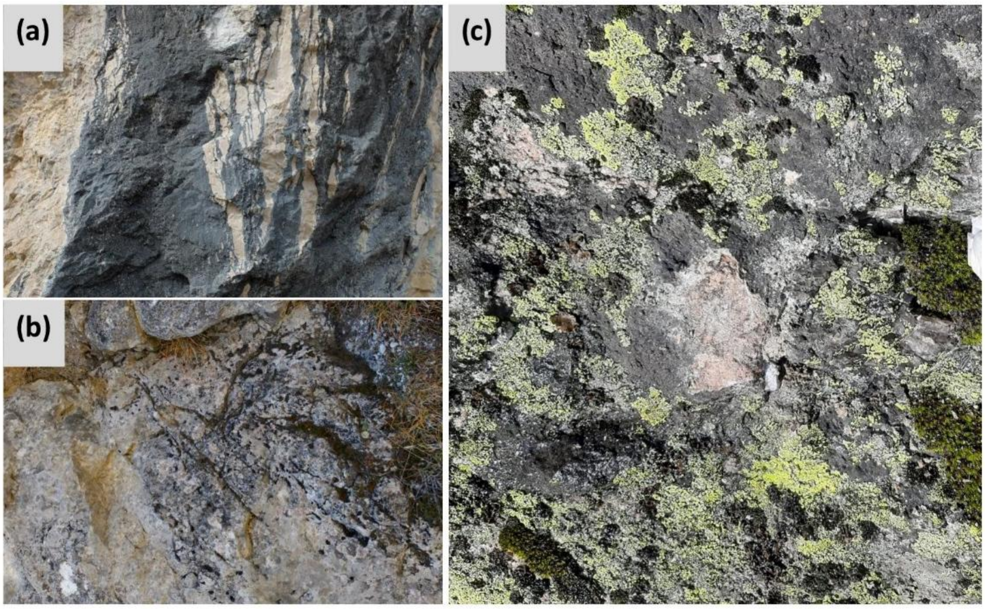

Figure S1. Examples of *Tintenstrich* microhabitats. **(a)** Contiguous *Tintenstrich*; **(b)** Mixed *Tintenstrich*; **(c)** Highly fragmented *Tintenstrich* - after Pittino, F.; Oliveira, J.; Fink, S.; Janssen, E.; Scheidegger, C. Lithic Bacterial Communities: Ecological Aspects Focusing on Tintenstrich Communities. *Front. Microbiol.* **2024**, *15*, 1430059. https://doi.org/10.3389/fmicb.2024.1430059.

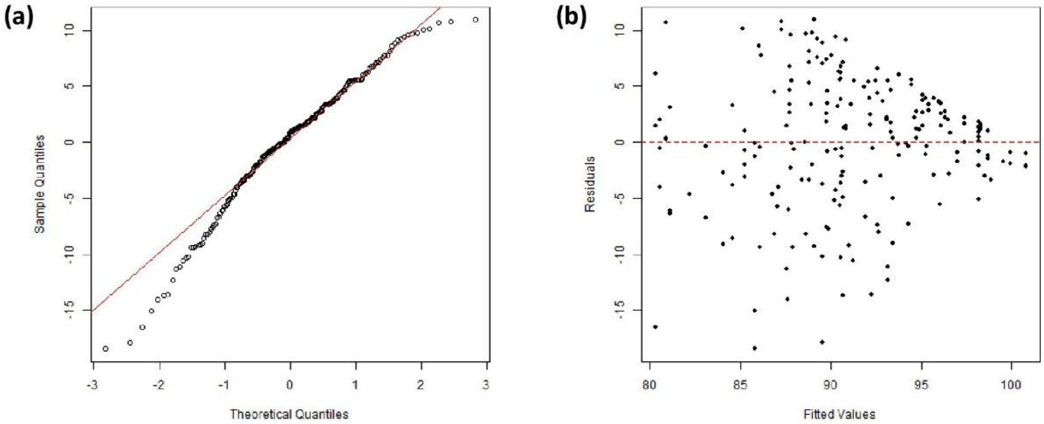

Figure S2. Diagnostic plots for Linear Mixed Models (LMMs) applied on cyanobacterial phyla abundance in all 207 *Tintenstrich* samples **(a)** Quantile-Quantile (Q-Q) plot demonstrating the distribution of observed versus expected residuals. A close alignment along the diagonal line indicates that the residuals follow a normal distribution, while deviations suggest potential non-normality in the residuals. **(b)** Residuals vs. Fitted plot showing the relationship between residuals and predicted values. Red line represents a smoothed trend (LOESS curve), indicating potential patterns in residuals. A LOESS curve suggests well-distributed residuals.

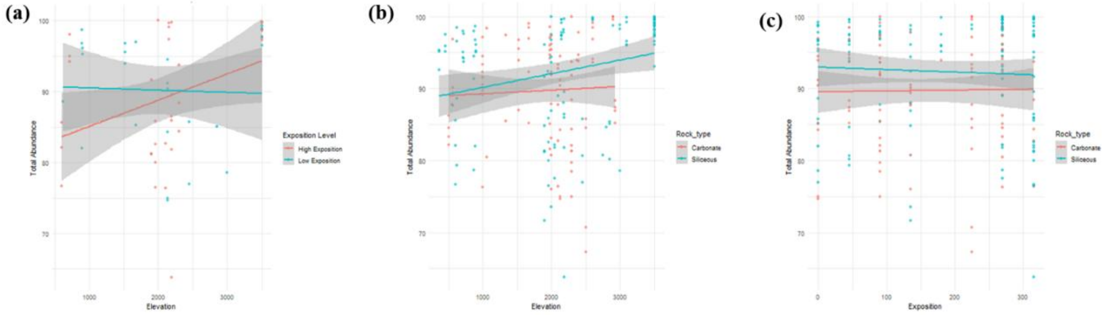

Figure S3. Visualizations depict the effects of environmental variable interactions on cyanobacterial abundance based on linear mixed-effects models (LMMs). **(a)** Interaction between elevation and exposure; **(b)** Interaction between siliceous rock type and elevation; **(c)** interaction between siliceous rock type and exposition.

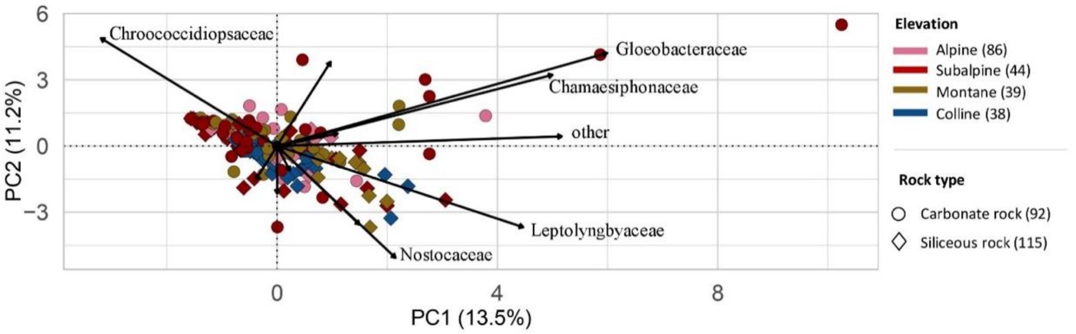

Figure S4. Principal Component Analysis (PCA) was performed on family-level data across all 207 *Tintenstrich* samples. PCA loadings represent the average abundance of each family per sample. Data points are color-coded to indicate the elevation at which samples were obtained, while different shapes denote rock types.

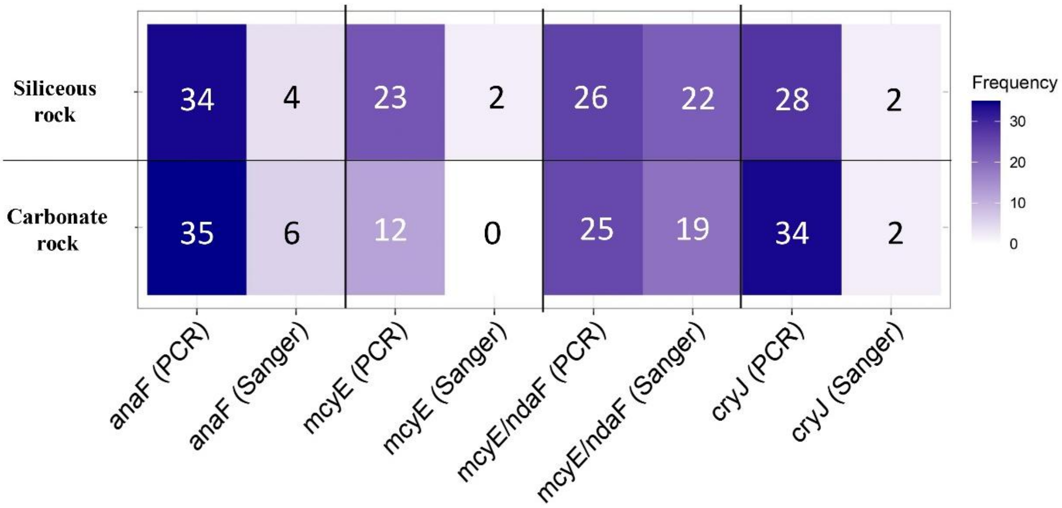

Figure S5. Matrix for toxin-encoding genes detected in *Tintenstrich* samples, comparing results obtained by end-point PCR and Sanger sequencing. The indicated values represent the number of samples in which toxin genes were detected, out of 207 samples analysed, categorized by rock type and target gene.
